# Navigating the field of implementation science towards maturity: challenges and opportunities

**DOI:** 10.1186/s13012-024-01352-0

**Published:** 2024-03-13

**Authors:** David A. Chambers, Karen M. Emmons

**Affiliations:** 1 Division of Cancer Control and Population Sciences, National Cancer Institute, 9609 Medical Center Drive, Room 3E-414, Rockville, Bethesda, MD 20850 USA; 2 Harvard T.H. Chan School of Public Health, Boston, MA USA

**Keywords:** Implementation science, Building capacity, Future work

## Abstract

**Background:**

The field of implementation science has significantly expanded in size and scope over the past two decades, although work related to understanding implementation processes have of course long preceded the more systematic efforts to improve integration of evidence-based interventions into practice settings. While this growth has had significant benefits to research, practice, and policy, there are some clear challenges that this period of adolescence has uncovered.

**Main body:**

This invited commentary reflects on the development of implementation science, its rapid growth, and milestones in its establishment as a viable component of the biomedical research enterprise. The authors reflect on progress in research and training, and then unpack some of the consequences of rapid growth, as the field has grappled with the competing challenges of legitimacy among the research community set against the necessary integration and engagement with practice and policy partners. The article then enumerates a set of principles for the field's next developmental stage and espouses the aspirational goal of a “big tent” to support the next generation of impactful science.

**Conclusion:**

For implementation science to expand its relevance and impact to practice and policy, researchers must not lose sight of the original purpose of the field—to support improvements in health and health care at scale, the importance of building a community of research and practice among key partners, and the balance of rigor, relevance, and societal benefit.

Contributions to the literature

- Implementation science has advanced significantly over the past few decades, with expanded capacity in research, training and funding opportunities across clinical, community and public health systems.
- This rapid growth has challenged the field to balance between integrating within biomedical research and ensuring that studies are relevant and responsive to practice and policy.
- The implementation science community should embrace the goal of a “big tent,” in which all partners can contribute to the development and execution of implementation studies.
- This goal may be aided by addressing a set of observations to support this community of science and practice.

## From whence implementation science came: the NIH experience and beyond

The roots of implementation science can be found in centuries past, with some dating back to the study of scurvy among sailors, maternal mortality in France, and the industrial revolution [1]. In more recent decades, it has had significant attention in the US (also referred to as Dissemination and Implementation Research), UK, and Canada (where it may be referred to as “Knowledge Translation”) [2–5], as well as other countries. While the full history of implementation science is beyond the scope of this article, we aim to show the trajectory of the field’s growth through a few selected examples.

In the US where we have done much of our work, several foundational efforts began to lay the groundwork for translation to practice, such as the articulation and use of the Diffusion of Innovation Model [6, 7], and community-based participatory research [8–10]. The acceleration of the developing field in the US and other countries was further catalyzed by both the increased focus on population health that came about in the 1980s [11] and the rise of Evidence-based Medicine in the 1990s [12–14] and the attention to the translation of research to practice that followed, through efforts by the Agency for Healthcare Research and Quality (the Translating Research Into Practice program), the Veterans Administration through its Quality Enhancement Research Initiative (QUERI), and the articulation of a field of dissemination and implementation research that the National Institutes of Health (NIH) and other US Federal agencies used in calls for a new generation of studies to improve health care and population health [15–17].

At the NIH, parallel efforts in both mental health and cancer created new capacity for the field to respond to funding opportunity announcements, participate in technical assistance workshops and conferences, and cultivate new scientific review panels charged with advancing the science. At the National Institute of Mental Health, workshops to advance child and adolescent D&I research, collaborations with the Substance Abuse and Mental Health Services Administration on “Science and Service” initiatives, and a mental-health-specific program announcement expanded upon a limited portfolio of studies [18]. At the National Cancer Institute, partnerships with the Centers for Disease Control and Prevention, American Cancer Society, and Robert Wood Johnson Foundation on dialogues for dissemination [19], grant supplement opportunities to develop dissemination plans for cancer control interventions and surveillance data and portals, and efforts to train cancer control practitioners on strategies to integrate “research-tested interventions” into practice and policy paved the way for a broader articulation of key research questions, theories and frameworks, and research designs needed to study high priority D&I topics.

By 2005, these focused activities gave way to an NIH-wide effort to advance D&I research more broadly, with a plan for agency-wide program announcements, and a dedicated special emphasis panel to conduct peer review. Capacity building efforts took place at annual conferences, and joint activities around offering in-depth training to the extramural community, initially through the use of R25 grants, which culminated in the development of the Office of Behavioral and Social Science Research-led Training Institute for Dissemination and Implementation Research in Health (TIDIRH). TIDIRH was later expanded to other disease topics (Training Institute for Dissemination and Implementation Research in Cancer (TIDIRC) OpenAccess | Division of Cancer Control and Population Sciences (DCCPS)) and other countries (TIDIRH-Ireland, TIDIRH-Australia, etc). In 2011, the Patient Centered Outcomes Research Institute (PCORI) was created and included in its mission the dissemination and implementation of comparative effectiveness research within healthcare practices. Simultaneously, efforts in Canada, the UK, Australia, and other countries toward embedding implementation science and knowledge translation into national research programs built significant capacity toward a cohort of successful investigators and hundreds of studies in healthcare.

In recent years, there has been tremendous growth in implementation science around the globe, with public and private funders establishing priorities for studying a range of different health topics, from the enormous investment in ending the global HIV pandemic to the World Health Organization and the Global Alliance for Chronic Diseases embracing opportunities to optimize care for non-communicable diseases. Indeed, the attention to implementation science has led toward multiple national and international networks (e.g., Nigerian (and Central and West Africa) Implementation Science Alliance (NISA/CWISA); European Implementation Society, etc.), and additional training opportunities for the next generation of scholars (e.g., King’s College London’s Implementation Science Masterclass (Implementation Science Masterclass (kcl.ac.uk)); NISA’s Public Health Research Week). In 2022, a Lancet Commission on Evidence-Based Implementation in Global Health was launched [20], to assess the state of implementation evidence in global health and develop an action plan for how to advance it in the coming years.

At the NIH, there have now been 18 years of flagship program announcements, an annual conference that is now in its 16th year, and over a decade of training programs supporting investigators interested in implementation science across health topics in clinical and community settings. We have seen the integration of implementation science into high-priority areas across the Agency (e.g., Cancer Moonshot, Rapid Acceleration of COVID Diagnostics for Vulnerable Populations (RADx-UP), The Helping to End Addiction Long-term® Initiative (NIH HEAL), the Transformative Research to Address Health Disparities and Advance Health Equity initiative), and a large number of health/disease topic-specific initiatives launched by Institutes, Centers, agencies and other funding organizations. Large-scale trials, research centers, career development awards, fellowships, and degree programs have all expanded over the past decade. Notably, these efforts have crisscrossed the globe, with even greater attention paid to global implementation science as a result of the COVID-19 pandemic, along with longstanding contributions of researchers and practitioners’ work in LMICs [21]. Many of the researchers and practitioners in this space have led the way in advancing theories, models, and frameworks (http://www.dissemination-implementation.org), categorizing implementation strategies [22], developing measures and methods [23–25], and identifying emerging opportunities for research [26].

## Reflections on the recent NIH investments in implementation science

### Research

As the field has grown and funding opportunities have been established to support implementation science studies, there have been several portfolio reviews that have assessed the extent to which NIH is supporting research in this space. Looking broadly across all of NIH, Purtle [27] reported that between 2007 and 2014, 146 studies were funded through the D&I funding announcements. NCI and NIMH each held roughly 30% of the funded D&I grants, followed by NIAD (12%) and NIDA (8%). This portfolio review also assessed the status of research on policy dissemination and implementation; only 8% of funded D&I grants across NIH were policy-focused. A more recent portfolio review of R01s that were reviewed by the Dissemination and Implementation Research in Health study section identified 84 funded R01s (2014–2016), 90% of which included implementation outcomes [28]. The majority of projects were conducted in clinical settings (e.g., acute or chronic care hospitals, clinics; 51.3%) or community settings (e.g., community organizations, workplaces, schools; 38.6%).

Reflecting NCI’s interest in supporting implementation science, there have been three portfolio reviews to determine the extent to which NCI is supporting research in this space. A review of funded studies through 2012 [29] identified 67 NCI grant awards having an implementation science focus, with significant growth from 2003 to 2012, albeit still a very small number of awards (4 and 15 total awards, respectively). Grants focused on cancer prevention were most common, while those targeting cancer treatment were least common. The authors noted a need for greater focus on measures development, assessment of how conceptual frameworks and their constructs lead to improved dissemination and implementation outcomes, and harmonization of rigorous yet pragmatic measures that can be used in multiple settings. An update of this portfolio review in 2021 [30] revealed only a slight amount of growth—71 grants funded— with more focus across the broader cancer continuum. The authors highlighted that relatively few grants studied sustainability, scale-up, de-implementation, or measure development. A recent study also evaluated NCI-funded research specifically in the cancer center setting [31]. Across the 64 comprehensive cancer centers, there were 74 active NCI-funded D&I research grants as of early 2021; 42% of comprehensive cancer centers had no NCI-funded D&I grants. As topics shift over time, additional portfolio analyses will be needed to continually evaluate current research priorities.

There have also been efforts to understand the extent to which the extant research is addressing key aspects of implementation science, and use of implementation science in specialty fields. Johnson and colleagues’ portfolio review [28] found that 67% of funded grants that were reviewed by the DIRH study section made references to sustainability, although none referred to sustainability planning. Few of the studies reviewed referenced frameworks with sustainability constructs or offered information on how they operationalized frameworks. This review illustrated the need for the field to better operationalize and test sustainability frameworks, and to develop strategies that will advance our understanding of how to maximize sustainability within implementation science.

Another key area in the field is de-implementation, or the process of “stop[ping] or reduc[ing] the use of inappropriate health interventions” [32]. Norton et al. [33] conducted a portfolio analysis of funded NIH and AHRQ grants focusing on de-implementation between 2000 and 2017. Across both agencies, there were only 20 grants funded with a focus on de-implementation over this 17-year period, studying both acute and chronic conditions but mostly focused on treatment. Forty percent of the grants focused on cancer, 10% on mental health, and 15% on infectious diseases. This review highlighted the need to increase research focused on the de-implementation of ineffective, unproven, and/or low-value health services.

Other portfolio reviews have highlighted the lack of an implementation science focus in genomics, a growing area of medicine for which there is a need to ensure widespread access across care settings. Roberts et al. [34] found that only 1.75% of investigator-initiated genomic grants funded by NIH between 2012 and 2016 had an implementation science focus. Further, these grants did not draw on implementation science frameworks, and most examined uptake of genomic medicine and/or assessed patient-centeredness rather than more standard implementation science methods or outcomes. Senier and colleagues [35] recently published a conceptual framework for genomic screening for high-risk hereditary conditions that merges insights from implementation science and sociological research on health inequities. This is an excellent effort to begin to consider how implementation science could contribute to the more equitable distribution of the benefits of genomic medicine.

As the field of implementation science has grown, there has been ubiquitous growth of high-level understanding of the importance of IS for translational science. We have seen a number of field-specific primers to IS published, designed to introduce researchers and practitioners to basic IS principles in fields as diverse as primary care [36], dermatology [37], nutrition [38], anesthesiology [39], and dentistry [40]. More in-depth engagement in IS has been led by the activities of researchers engaged in the NIH Clinical Translational Science Awards (CTSAs). There has been an important emphasis on the application of implementation science throughout the translational continuum and particularly to early-stage research [41–43]. Recognizing the tremendous potential to support the advancement and impact of D&I science across the translational continuum, Shelton et al. [44] surveyed the 67 funded CTSAs; 43 reported delivering D&I research services. Among those with a D&I resource, challenges faced included an inadequate D&I science workforce and limited understanding of D&I science. Recommendations included that efforts be made to increase training to meet demand and to expand the workforce, that more accessible D&I tools/resources be created, and that there be greater visibility and awareness of D&I methods. Notably, the most recent CTSA FOA (PAR-21-293) had a significant emphasis on development of capacity and infrastructure for use of IS.

### Training

Recognizing both that few graduate schools provide an in-depth curriculum in implementation science and the importance of grounding investigators in the field of implementation science, several training programs have been developed and evaluated, as noted above. The most robust programs have been in mental health and cancer.

NIMH began funding the Implementation Research Institute (IRI) in 2009, with an emphasis on growing the intellectual knowledge base of implementation science as applied to mental health topics. The intensive two-year training for approximately 10 new fellows each year focuses on encouraging scholarly productivity, mentorship, grant-writing, and assisting established researchers who are interested in transitioning into implementation science [45]. Of the 53 IRI graduates between 2011 and 2016, 62% subsequently received NIH, VA, and PCORI research awards [46].

The NIH Training Institute for Dissemination and Implementation Research in Health (TIDIRH) was launched in 2011 by OBSSR, in collaboration with NCI and NIMH, and was subsequently broadened through partnerships with other institutes and the VA. TIDIRH largely focused on researchers at earlier stages of career development (i.e., post-doctoral), and evolved from a residential weeklong training program into an extended 3–4 month hybrid program with online and multi-day in-person training to assist researchers in preparation and submission of NIH applications [47] (Meissner et al. 2013). Over its first 5 years, TIDIRH provided an in-person, week-long training to 197 investigators who were new to the dissemination and implementation (D&I) research field. A major goal was to build the field, at least in part through networking and collaboration. Vinson et al. [48] conducted an evaluation of the program, comparing trainees with unselected applicants (UAs) whose application score was within one standard deviation of the mean for trainees’ scores in the same application year. TIDIRH trainees submitted more peer-reviewed NIH grants per person than UA and had significantly better funding outcomes. Metrics related to collaboration and networking suggested that the program met its goals to create a scientific community in implementation science.

Implementation science has been a particular focus of NCI. A cancer-focused version of TIDIRH, the Training in Dissemination and Implementation Research in Cancer (TIDIRC) has been offered since 2018, and all materials are now available in open access format. Building on the more intensive mentoring approach offered by IRI, the Mentored Training in Dissemination and Implementation Research in Cancer (MT-DIRC) was developed in 2014 using the R25 mechanism. Brownson et al. [49] used a quasi-experimental design to compared changes in MT-DIRC fellows across multiple competencies. Fellows' self-rated skills in D&I competencies improved significantly in all domains over time. Mentorship and collaboration networks related to development of manuscripts and research also grew over time.

Training grant mechanisms have also been used broadly within the cancer community to support implementation science training. In 2021, 30 out of 64 comprehensive cancer centers had a D&I-focused training grant; 9 were institutional R25/T32 grants [31]. Another emerging model is the development of time-limited, intensive in-person training efforts designed by NIH-funded grantees, with a focus on building capacity (e.g., the Implementation Science Institute (ISI) at the University of Pennsylvania; the Summer Institute on Implementation Science at the University of North Carolina; the Intensive Course in Fundamentals of Implementation Science in Global Health at the University of Washington; IMPlementation to Achieve Clinical Transformation (IMPACT) at the University of Colorado [50]. Similar efforts have been reaching implementation scientists globally, including a Massive Open Online Course (MOOC) on implementation research in infectious diseases (https://tdr.who.int/home/our-work/strengthening-research-capacity/massive-open-online-course-(mooc)-on-implementation-research) and many workshops and courses held in multiple regions.

### Summary of current state

The field has observed significant growth in training opportunities, although the continued integration of IS into major NIH initiatives suggests that training needs still outpace the available opportunities, especially for the highly mentored programs that have demonstrated their ability to improve access to IS mentoring, increase scientific productivity, and improve networking and collaboration. Although there has been growth in research funding of implementation science studies, the number of funded studies is still quite modest. The level of engagement of implementation science infrastructures has also grown, although 42% of comprehensive cancer centers have no NCI-funded D&I grants, and over one-third of CTSAs do not offer any D&I research services.

## Consequences of rapid growth

There have recently been several commentaries and critiques of the field of implementation science that take stock of the gaps in the field that threaten to limit its impact. We would argue that several of the issues raised are a consequence of the rapid and pervasive growth of the field. For example, Beidas et al. used a pre-mortem approach to identify issues that threaten the field’s forward movement if not addressed in the context of its evolution. Three themes identified are related to the speed and way in which the field has grown. First is the over-emphasis on becoming a “legitimate” science, manifest as explosive development of theoretical frameworks and lack of critical thinking about selection of implementation frameworks and outcomes for the specific issue and setting being studied. As a result, there is a surface-level of understanding and engagement in the framework, without leveraging the full body of knowledge available. Second is the role that implementation scientists have played in serving as gatekeepers, as they seek to establish the field’s coherence. This has limited the deep engagement of investigators who have had limited training, and reinforcement of surface-level approaches. The trainings that are available are generally one-size-fits-all, and do not consider the differing levels of training that investigators might need, assuming that not all are looking to become full-time implementation scientists. Indeed, others have noted the relatively limited emphasis on capacity building, both in terms of the investigator community and implementation partners [51–54]. Third, as the field has developed it has focused on interventions that are relatively easy to implement, largely in health care settings, such as increasing uptake of well-accepted screenings and preventive interventions that are feasibly delivered broadly and have often chosen sites that have sufficient resources to deliver a range of different services. There has been little consideration of structural factors that are critical to population health, and as result, our actual impact on health equity may be limited. Further, metrics used to judge the success of implementation studies (e.g., publications, grants) likely do not reflect population impact, instead focusing on traditional measures of scientific impact [55].

Wensing and Grol [56] also note several challenges that have limited the contribution of implementation science to knowledge translation that we contend are related to the field’s rapid growth. They argue that the fit between implementation problems and solutions is often limited by the implementation teams’ professional background, rather than the fit of the solution to the problem. This is likely a function of the team being familiar with one or two approaches through introductory trainings, rather than deep immersion in a broader set of tools. Another issue is the proliferation of research that is descriptive in nature, with limited testing and validation efforts, particularly across frameworks and models. This has similarly led to a limited focus on development of robust outcome measures, and over-reliance on measures with uncertain psychometric properties. And finally, although stakeholder engagement is a critical component of implementation science, there have been limited efforts to refine and validate rigorous methods of engagement.

We would argue that there are additional issues that have resulted from the field’s rapid growth that should be considered if it is to mature into a robust and impactful science. For example, the research and practice communities remain largely siloed, with relationships developed at the point of implementation opportunity, rather than development of strong partnerships that then lead to collaboration. The Implementation Science Centers in Cancer Control are utilizing a different model, in which implementation “laboratories,” or partners are built and resourced, and a range of collaborative implementation studies conducted over time [57]. This is a direct relative of the longstanding concept of the practice-based research networks, built over decades in primary care and other clinical and community settings (e.g., NAPCRG’s PBRN meeting). Another major issue is that, due to the recognition of the field’s potential contribution and inclusion in several funding opportunities, demand for expertise exceeds availability. This has led to a “self-study” approach, which, while much needed and laudable, may limit the depth of understanding that can be brought to bear. Further, efforts to train investigators have largely been organized by disease topic due to funding models, which has left investigators in some fields with little choice but to learn what they can independently. A considerable concern is that our training programs need to evolve, both to be more inclusive as well as to move beyond the beginner and intermediate level, so that as the field evolves the training opportunities deepen. It is also important to consider how to build IS as a fundamental research skill in the most efficient way. Should graduate training programs be expected to offer required courses in implementation science as part of their methods curricula, or to at least offer an elective course sequence in implementation science?

There has further been criticism about the field’s rapid expansion in its own silo, and that the pace of its development has led to co-opting of strategies identified by other fields (e.g., participatory research; quality improvement) without recognizing existing efforts [58]. This limits the development of those strategies and risks re-creation of similar concepts under new names. This has further contributed to the terminology creep that we have seen as the field has expanded. In fact, there is so much variation in definitions used in the field that efforts have been undertaken to create glossaries [59]. As the field develops, it is important that we are less diffuse in our definition of terminology, and that we build on other fields rather than re-inventing or re-naming well-developed concepts.

Although we applaud the efforts of funders to require use of implementation science approaches, there is a risk if funders do not fully consider what is required. As noted above, effective implementation requires partnerships with practice communities and settings, and participation of practitioners requires resources, in the same way it does for researchers. Research funding mechanisms that don’t ensure inclusion of community resources are unlikely to achieve their full potential.

## Advancing implementation science into a new generation—creating a big tent

As implementation science makes its way beyond the challenges of adolescence and into young adulthood, it will serve us well to reset some of the assumptions from prior days. While we recognize that building the scientific integrity of the field was important to drive early conceptual and empirical advances, we must redouble our efforts around opening our doors to all who are interested in improving (and affected by) health and healthcare. Here, we lay out 5 key observations that may help shape the field in its next decade of development.

### Observation one: implementation science is about relevant, impactful questions, and robust, rigorous, and valid research methods and measures, not identities or terms

The formalization of implementation science as an academic discipline succeeded in raising its valence, spotlighting the gaps in knowledge to support the full translational pathway, and enabled collectivity among researchers who felt isolated within the traditional biomedical research paradigm. Consequently, in creating an identity and specific terms and definitions for the field, it established both an “in” and an “out” lens to describe each individual’s relationship to implementation science. This may have been especially alienating to those who worked in adjacent fields (e.g., quality improvement; improvement science) that did not align exactly with emerging content, but that had robust methods that had been widely adopted within health care. In essence, this created a dynamic that is at odds with the goal of a big tent beyond boundaries. To move beyond this, we must reorient towards simple principles—that the research questions being asked are more important than researchers identifying themselves by specific disciplinary labels, and the approaches we use to answer those questions are more important than terms of what is and what is not an implementation study. As part of this reorientation, we see the value in studying the full range of interventions, programs, and practices being implemented, beyond those categorized as “evidence-based”, as the strategies supporting their adoption, implementation, and sustainment can decidedly add to our D&I knowledge base. Furthermore, we note the ongoing need to balance the rigor of study designs with what is most feasible and relevant to conduct within the context of clinical and community settings, as well as ensure that the information generated is timely for practice and policy.

### Observation two: implementation science is built on a foundation of medical, behavioral, and social sciences and values and leverages those contributions

The multi-disciplinary, team-based approach to implementation science is well-established, and yet there are tensions as to whether implementation science seems to be “reinventing” established theories, measures, and empirical data. As mentioned above, there may have been incentives to establish the uniqueness of implementation science as a field as a way to justify ongoing efforts. This may have led to tensions and minimization of the significant contributions that so many fields have made to our current dissemination and implementation knowledge base and in the long run, will impede progress toward effectively utilizing all that we know to address what we do not. Leveraging the many relevant streams of knowledge enables us to build upon prior knowledge rather than perceptions of rediscovering what is already known. Acknowledgment of the foundation upon which implementation science is based in no way diminishes its added value—it simply celebrates the historic contributions to our theoretical and empirical knowledge base. It also may have the added benefit of enabling a more critical understanding of our TMFs and how they can be most helpful in generating and testing hypotheses, and in advancing methods and measures.

### Observation three: support and capacity building is needed for all implementation science-oriented partners, at increasing levels of depth

As noted, over the past fifteen or so years the field has done a wonderful job of creating many opportunities for those new to implementation science to get a deep introduction into the constituent parts of implementation science (e.g., frameworks, outcomes, strategies, methods) and to receive guidance around specific study concepts. While success has come for research-focused trainees in receiving grants, publishing papers, and landing positions at research organizations as implementation scientists, much less has been done to support ongoing growth for the field. An expanded capacity-building approach is needed that articulates and creates ongoing pathways for mentoring beyond the ”100-level courses” and establishes sustainable networks of investigators and community and system partners to foster longer-term support. Although initial efforts to explicate implementation science competencies did identify a few advanced skills (e.g., de-adoption and de-implementation; application of economic evaluation to implementation studies; scale-up and spread methodologies), articulated competencies are largely at the beginner and intermediate levels [60]. Given the growth of the field since these competencies were identified, it may be time to re-think what now constitutes advanced skills and ensure that there are pathways for enhanced training in these areas. It is also critical to expand our capacity building beyond investigators and to ensure high-quality and in-depth training is available for practice and policy partners as well.

### Observation four: an implementation science ecosystem, made up of partnerships among community and clinical settings, is essential to scaling up what we ask and what we learn

The recognition of practice-based research networks in advancing relevant, impactful improvements to health and health care preceded the recent expansion of implementation science. The articulation and establishment of implementation laboratories [4, 61] to support ongoing assessment and improvement of implementation activities has become a focus of multiple, national funding initiatives. This lays the foundation for a larger set of partnerships within service settings that can coalesce into an “implementation science ecosystem,” by which common data elements to track implementation can be captured by investigators and partners, local success in implementation can be scaled up across networks, and learning from longer-term initiatives (e.g., studying sustainment, de-implementation, adaptation and evolution of EBPs) can be achieved. This ecosystem is an articulation, described below, of a question-focused approach to implementation science, in which we consider how to better synthesize what we learn, identify key gaps in knowledge, and support efficient ways to overcome those gaps. It offers a safe space for experimentation, under the guidance of our key clinical and community partners. Naturally, such an approach cannot be advanced without key resources, so careful consideration of necessary inputs and their source will be required.

### Observation five: patients, practitioners, and policymakers should be at the center of implementation science investigations

Relatedly, to ensure that our field is asking the right questions about adoption, implementation, sustainment, de-implementation, and other priority areas, we need to make explicit efforts to keep the most important partners (patients, communities, practitioners, and policymakers) at the center of our work. This means ongoing conversations to identify the most important questions related to implementation efforts that will drive decisions of patients/people to improve their health, practitioners to optimize their practice, and policymakers to create the necessary conditions where optimal care becomes standard. One can imagine an IS information system that solicits ongoing suggestions from partners about what to study, captures feedback on how prior studies have effectively engaged partners, and synthesizes bodies of knowledge that support explicit decisions made from individual to societal levels.

To actualize this vision, it may be useful as a field to consider what resources are needed going forward (see Table 1). We argue that the field should move beyond the introductory training courses to consider lifelong learning and mentoring support across the career trajectory. In order to meet this charge, there are several “Wh-“ questions that must be answered that articulate the parties involved (who), the targets (what), settings (where), outcomes (why), timing (when), and approach (how). For example, we should re-think our sometimes siloed training programs that focus on a single disease entity or field, and shift to broad training that includes all implementation partners and evidence users. Cross-field training would likely maximize learning and that the learning would lead to action. This would also help implementation science to more seamlessly integrate within policy, practice, and research worlds, avoid being siloed off from practice and policy communities toward scientific legitimacy, while also benefitting from relevant research advances from basic to translational to clinical to population science. We should also consider what approaches can we take among research-practice partnership teams to ensure that we are prioritizing the most impactful questions and how can we address cross-cutting issues, beyond the disease-siloed approach that we typically take in our inquiries. For example, we could learn significantly by considering questions of adaptation or sustainment across settings and topic areas. As we expand our engagement efforts, we should consider the full range of potential implementation settings, moving beyond those that have a history of engagement in research and build new collaborations with partners that can bring new perspectives and enable evidence to reach those who have had more limited benefit from evidence-based approaches. Shifting from the identification of research questions by funding agencies and researchers to collaborative questions raised in real time by partners would also likely improve the outcomes of our efforts.

**Table 1:** Articulating the “WH-” and “How” underlying where we implementation science to be in the future and example strategies toward getting us there

	Current state	Vision	Strategies	Relevant observation
Who	• Research-focused training • Trainees often from one or only a few fields	• Training that includes all implementation partners (e.g., implementation scientists, practitioners, policymakers, community organizations, health systems) • Trainings cross fields to maximize learning	• Engagement efforts • Training of researchers, practitioners, and policymaker teams • Solicitation of key priorities of all	#3: Support and capacity building is needed for all Implementation Science (IS)-oriented partners, at increasing levels of depth. #4: An IS Ecosystem, made up of partnerships among community and clinical settings, is essential to scaling up what we ask and what we learn. #5: Patients, practitioners, and policymakers should be at the center of IS investigations.
What	• Primary focus on applying IS to initial implementation as a single point in the IS lifecycle • Mix of virtual and in-person training	• High priority areas of study • Adaptation/evolution/sustainment • Bundling of EBPs • De-implementation • Sustainment • Develop empirical evidence about the benefits of different training formats for different skill levels.	• Workshops and conferences • Ongoing funding opportunities	#1: IS is about relevant, impactful questions, and robust, rigorous, and valid research methods and measures, not identities or terms. #2: IS is built on a foundation of medical, behavioral, and social sciences and values and leverages those contributions. #4: An IS Ecosystem, is essential to scaling up
Where	• Opportunistic in selection of setting • Partnerships in a limited range of settings • Partners typically have a history of research engagement	• Broader diversity of implementation settings and partners • Partnerships selected strategically and holistically	• Engagement of system/community leaders to support integration in a range of settings • Exploration/enhancement of existing data systems • Assess fit with ongoing activities (e.g., QI, implementation efforts)	#4: An IS ecosystem, is essential to scaling up #5: Patients, practitioners, and policymakers should be at the center of IS investigations.
Why	• To implement a specific intervention in a specific setting	• To provide optimal clinical and community care to all	• Identify metrics of optimal care across settings • Develop dashboard to support prioritization	#4: An IS Ecosystem, is essential to scaling up #5: Patients, practitioners, and policymakers should be at the center of IS investigations.
When	• Researchers identify questions when funding opportunities arise	• As questions arise and answers are needed by partners	• Improve rapid cycle methods of research • Improve data sharing/pooled analyses	#1: IS is about relevant questions, and rigorous methods and measures, not identities or terms. #4: An IS Ecosystem, is essential to scaling up #5: Patients, practitioners, and policymakers should be at the center of IS investigations.
How	• Research team identifies topic and questions of interest • Design choices focus on rigor	• Data analysis yields gaps/inequities in implementation • Questions are developed and prioritized by teams • Range of designs to be responsive, rigorous, and relevant to context • Findings are shared across the “tent”	• Creation of an implementation science data ecosystem • Collaborative platforms to support question generation and prioritization • Availability of resources to support use of rigorous and relevant study designs	#1: IS is about relevant questions, and rigorous methods and measures, not identities or terms. #2: IS is built on a broad foundation and values and leverages those contributions #3: Support and capacity building is needed for all partners, at depth #4: An IS Ecosystem, is essential to scaling up #5: Patients, practitioners, and policymakers should be at the center of IS investigations.

## Conclusions

The ultimate aim for these observations is to re-establish implementation science as a “big tent,” building upon a foundation of the many fields that implementation science has learned from in creating its theoretical and empirical base. The capacity of the field relies on the “tentpoles” of continued expansion of training opportunities, capacity to conduct studies and support implementation activities across a broad range of service contexts, and the advancement of methods and measures to optimally and responsively design and execute relevant and impactful studies. The partnerships are expected to be dynamic and expanding over time, with teams of multiple disciplines and vantage points evolving according to need and capacity as they identify high priority questions and the best solutions to our implementation challenges. Ultimately, the bigger the tent and the more collaboration among those in the tent and harmonization of methods used, the larger the knowledge base that we can generate, can synthesize, and can utilize as we work to equitably improve implementation of effective health and healthcare for all.

As a field, we will be best served if we review the distance between an ideal vision of activity in implementation science and current activities and employ various strategies toward achieving the promise. There is no question that the enthusiasm and progress surrounding implementation science and the potential for its impact remains; the task at hand is to thoughtfully and strategically create that bright and impactful future. In doing so, we again envision implementation science as fulfilling the promise of balancing rigor, relevance, and societal benefit toward better health and health care for all.

## Data Availability

Not applicable.

## References

[1] Swanson JA, Schmitz D, Chung KC (2010). How to practice evidence-based medicine. Plast Reconstr Surg.

[2] Lomas J (1993). Diffusion, dissemination, and implementation: who should do what?. Ann N Y Acad Sci.

[3] Grimshaw J, Eccles M, Thomas R (2006). Toward evidence-based quality improvement. Evidence (and its limitations) of the effectiveness of guideline dissemination and implementation strategies 1966-1998. J Gen Intern Med..

[4] Grimshaw JM, Ivers N, Linklater S (2019). Reinvigorating stagnant science: implementation laboratories and a meta-laboratory to efficiently advance the science of audit and feedback. BMJ Qual Saf.

[5] Greenhalgh T, Robert G, Macfarlane F, Bate P, Kyriakidou O (2004). Diffusion of innovations in service organizations: systematic review and recommendations. Milbank Q.

[6] Rogers E (2003). Diffusion of Innovations.

[7] Dearing JWKF, Peng T-Q, Brownson RCG, Proctor E (2023). Historical Roots of Dissemination and Implementation Science. Dissemination and Implementation Research in Health.

[8] Wallerstein NB, Duran B (2006). Using community-based participatory research to address health disparities. Health Promot Pract.

[9] Wallerstein N, Duran B (2010). Community-based participatory research contributions to intervention research: the intersection of science and practice to improve health equity. Am J Public Health..

[10] Israel BA, Parker EA, Rowe Z (2005). Community-based participatory research: lessons learned from the Centers for Children's Environmental Health and Disease Prevention Research. Environ Health Perspect.

[11] Rose G (1985). Sick individuals and sick populations. Int J Epidemiol.

[12] Rosenberg WM, Sackett DL (1996). On the need for evidence-based medicine. Therapie.

[13] Sackett DL, Rosenberg WM (1995). The need for evidence-based medicine. J R Soc Med.

[14] Sackett DL, Rosenberg WM, Gray JA, Haynes RB, Richardson WS (1996). Evidence based medicine: what it is and what it isn't. BMJ.

[15] Glasgow RE (2013). What does it mean to be pragmatic? Pragmatic methods, measures, and models to facilitate research translation. Health Educ Behav.

[16] Glasgow RE, Klesges LM, Dzewaltowski DA, Bull SS, Estabrooks P (2004). The future of health behavior change research: what is needed to improve translation of research into health promotion practice?. Ann Behav Med.

[17] Glasgow RE, Lichtenstein E, Marcus AC (2003). Why don't we see more translation of health promotion research to practice? Rethinking the efficacy-to-effectiveness transition. Am J Public Health.

[18] Chambers DA (2008). Advancing the science of implementation: a workshop summary. Adm Policy Ment Health.

[19] Kerner JGR, Vinson CA, DA VC C, Norton WE (2018). A history of the national cancer institute’s support for implementation science across the cancer control continuum: Context counts. Advancing the Science of Implementation Across the Cancer Continuum.

[20] Peterson HB, Dube Q, Lawn JE, Haidar J, Bagenal J, Horton R (2023). Achieving justice in implementation: the Lancet Commission on Evidence-Based Implementation in Global Health. Lancet.

[21] Theobald S, Brandes N, Gyapong M (2018). Implementation research: new imperatives and opportunities in global health. Lancet.

[22] Powell BJ, Waltz TJ, Chinman MJ (2015). A refined compilation of implementation strategies: results from the Expert Recommendations for Implementing Change (ERIC) project. Implement Sci.

[23] Rabin BA, Purcell P, Naveed S (2012). Advancing the application, quality and harmonization of implementation science measures. Implement Sci.

[24] Fernandez ME, Walker TJ, Weiner BJ (2018). Developing measures to assess constructs from the Inner Setting domain of the Consolidated Framework for Implementation Research. Implement Sci.

[25] Brown CH, Curran G, Palinkas LA (2017). An Overview of Research and Evaluation Designs for Dissemination and Implementation. Annu Rev Public Health.

[26] Brownson RC, Kumanyika SK, Kreuter MW, Haire-Joshu D (2021). Implementation science should give higher priority to health equity. Implement Sci.

[27] Purtle J, Peters R, Brownson RC (2016). A review of policy dissemination and implementation research funded by the National Institutes of Health, 2007-2014. Implement Sci.

[28] Johnson AM, Moore JE, Chambers DA, Rup J, Dinyarian C, Straus SE (2019). How do researchers conceptualize and plan for the sustainability of their NIH R01 implementation projects?. Implement Sci.

[29] Neta G, Sanchez MA, Chambers DA (2015). Implementation science in cancer prevention and control: a decade of grant funding by the National Cancer Institute and future directions. Implement Sci.

[30] Neta G, Clyne M, Chambers DA (2021). Dissemination and Implementation Research at the National Cancer Institute: A Review of Funded Studies (2006-2019) and Opportunities to Advance the Field. Cancer Epidemiol Biomarkers Prev.

[31] Mueller NM, Hsieh A, Ramanadhan S, Lee RM, Emmons KM (2022). The Prevalence of Dissemination and Implementation Research and Training Grants at National Cancer Institute-Designated Cancer Centers. JNCI Cancer Spectr.

[32] Norton WE, Chambers DA, Kramer BS (2019). Conceptualizing De-Implementation in Cancer Care Delivery. J Clin Oncol.

[33] Norton WE, Kennedy AE, Chambers DA (2017). Studying de-implementation in health: an analysis of funded research grants. Implement Sci.

[34] Roberts MC, Clyne M, Kennedy AE, Chambers DA, Khoury MJ (2019). The current state of funded NIH grants in implementation science in genomic medicine: a portfolio analysis. Genet Med.

[35] Senier L, McBride CM, Ramsey AT, Bonham VL, Chambers DA (2019). Blending Insights from Implementation Science and the Social Sciences to Mitigate Inequities in Screening for Hereditary Cancer Syndromes. Int J Environ Res Public Health.

[36] Bazemore A, Neale AV, Lupo P, Seehusen D (2018). Advancing the Science of Implementation in Primary Health Care. J Am Board Fam Med.

[37] Ashrafzadeh S, Metlay JP, Choudhry NK, Emmons KM, Asgari MM (2020). Using Implementation Science to Optimize the Uptake of Evidence-Based Medicine into Dermatology Practice. J Invest Dermatol.

[38] Murofushi K, Badaracco C, County C (2021). Implementation Science in Evidence-based Nutrition Practice: Considerations for the Registered Dietitian Nutritionist. J Acad Nutr Diet.

[39] Lane-Fall MB, Cobb BT, Cené CW, Beidas RS (2018). Implementation Science in Perioperative Care. Anesthesiol Clin.

[40] Frantsve-Hawley J, Kumar SS, Rindal DB, Weyant RJ (2020). Implementation science and periodontal practice: Translation of evidence into periodontology. Periodontol 2000.

[41] Leppin AL, Mahoney JE, Stevens KR (2020). Situating dissemination and implementation sciences within and across the translational research spectrum. J Clin Transl Sci.

[42] Mehta TG, Mahoney J, Leppin AL (2021). Integrating dissemination and implementation sciences within Clinical and Translational Science Award programs to advance translational research: Recommendations to national and local leaders. J Clin Transl Sci.

[43] Mehta TG, Mahoney J, Leppin AL (2021). Erratum: Integrating dissemination and implementation sciences within Clinical and Translational Science Award programs to advance translational research: Recommendations to national and local leaders - ERRATUM. J Clin Transl Sci.

[44] Shelton RC, Dolor RJ, Tobin JN (2022). Dissemination and implementation science resources, training, and scientific activities provided through CTSA programs nationally: Opportunities to advance D&I research and training capacity-ADDENDUM. J Clin Transl Sci.

[45] Proctor EK, Landsverk J, Aarons G, Chambers D, Glisson C, Mittman B (2009). Implementation research in mental health services: an emerging science with conceptual, methodological, and training challenges. Adm Policy Ment Health.

[46] Chambers DA, Pintello D, Juliano-Bult D (2020). Capacity-building and training opportunities for implementation science in mental health. Psychiatry Res..

[47] Meissner HI, Glasgow RE, Vinson CA (2013). The U.S. training institute for dissemination and implementation research in health. Implement Sci.

[48] Vinson CA, Clyne M, Cardoza N, Emmons KM (2019). Building capacity: a cross-sectional evaluation of the US Training Institute for Dissemination and Implementation Research in Health. Implement Sci.

[49] Brownson RC, Jacob RR, Carothers BJ (2021). Building the Next Generation of Researchers: Mentored Training in Dissemination and Implementation Science. Acad Med.

[50] Huebschmann AG, Johnston S, Davis R (2022). Promoting rigor and sustainment in implementation science capacity building programs: A multi-method study. Implement Res Pract.

[51] Ramanadhan S, Aronstein D, Martinez-Dominguez V, Xuan Z, Viswanath K (2020). Designing Capacity-Building Supports to Promote Evidence-Based Programs in Community-Based Organizations Working with Underserved Populations. Prog Community Health Partnersh.

[52] Ramanadhan S, Mahtani SL, Kirk S (2022). Measuring capacity to use evidence-based interventions in community-based organizations: A comprehensive, scoping review. J Clin Transl Sci.

[53] Leeman J, Calancie L, Hartman MA (2015). What strategies are used to build practitioners' capacity to implement community-based interventions and are they effective?: a systematic review. Implement Sci.

[54] Leeman J, Calancie L, Kegler MC (2017). Developing Theory to Guide Building Practitioners' Capacity to Implement Evidence-Based Interventions. Health Educ Behav.

[55] Luke DA, Sarli CC, Suiter AM (2018). The Translational Science Benefits Model: A New Framework for Assessing the Health and Societal Benefits of Clinical and Translational Sciences. Clin Transl Sci.

[56] Wensing M, Grol R (2019). Knowledge translation in health: how implementation science could contribute more. BMC Med.

[57] Kruse GR, Hale E, Bekelman JE (2023). Creating research-ready partnerships: the initial development of seven implementation laboratories to advance cancer control. BMC Health Serv Res.

[58] Tabak RG, Padek MM, Kerner JF (2017). Dissemination and Implementation Science Training Needs: Insights From Practitioners and Researchers. Am J Prev Med.

[59] Rabin BB, RC., Brownson RCCG, Proctor EK (2018). Terminology for Dissemination and Implementation Research. Dissemination and Implementation Research in Health.

[60] Padek M, Colditz G, Dobbins M (2015). Developing educational competencies for dissemination and implementation research training programs: an exploratory analysis using card sorts. Implement Sci.

[61] Ivers NM, Grimshaw JM (2016). Reducing research waste with implementation laboratories. Lancet.

